# Obstructive sleep apnea domains: Knowledge, attitude and practice results of dentists from a dental college in India

**DOI:** 10.5935/1984-0063.20190121

**Published:** 2020

**Authors:** Sonal Sadashiv Kale, Pradnya Kakodkar, Sahana Hedge Shetiya

**Affiliations:** Dr. D.Y. Patil Vidyapeeth Pune, Dr. D.Y. Patil Dental College and Hospital Pimpri, Pune, Department of Public Health Dentistry - Pune - Maharashtra - India.

**Keywords:** Obstructive Sleep Apnea, Knowledge, Attitude, Practice Management, Dental

## Abstract

**Objective:**

To assess the knowledge, attitude and practice regarding different domains of obstructive sleep apnea (OSA) amongst dentists from a dental college in India.

**Methods:**

112 dentists participated in the study. A 23-item, self-designed, pre-tested and validated questionnaire assessing the knowledge, attitude and practice (KAP) regarding nine different domains was used to collect the data. Knowledge and practice was categorized domain wise as good/poor, while attitude as favorable/unfavorable.

**Results:**

Dentists were observed to have good knowledge about OSA, for domains concerning to the definition (60.71%), general findings (76.19%) and risk factors of OSA (66.96%). For the domain of screening and diagnosis (38.83%) along with treatment and referral (36.01%) dentists presented poor knowledge. 100% favorable attitude was reported for all the domains, while the dentists poorly faired (<50%) for both the practice domains.

**Conclusion:**

In spite of dentists showcasing favorable attitude towards OSA, they possessed poor knowledge for domains concerning screening, diagnosis and treatment modalities of OSA which may be linked to the hurdle in their way of practice. Thus a special attention towards these domains needs to be given so as to improve the handling skills of dentist for OSA patients coming to their clinics and prevent further health related issues.

## INTRODUCTION

Obstructive sleep apnea (OSA) is a sleep disorder characterized as complete cessation of breathing for 10 seconds or more during sleep due to complete or partial pharyngeal obstruction leading to frequent arousal during sleep and excessive day time sleepiness^1,2^.

The gold standard for diagnosing OSA is polysomnography^3^. But, due to its complicated diagnostic procedure and patient’s discomfort towards the procedure other diagnostic measures have been stated in the literature^4^. One of them is the use of questionnaire tool for OSA diagnosis. Many questionnaires have been developed to overcome this drawback like BANG, STOP-BANG, ARES, Berlin, Epworth Sleepiness Scale and STOP among adults^5^ and Pittsburg Sleep Quality Index^6^ for children. These questionnaires allow the practitioners to screen the patients and refer only the positively screened patients for final diagnosis through polysomnography, saving the time and cost of the patients.

The treatment of OSA depends on the severity of the condition. Whatever the mode of management is, it is very crucial to first screen the condition and attain a proper diagnosis.

OSA is considered to be a fatal disease as it has been linked with systemic diseases like hypertension, obesity and other cardiovascular diseases^7-9^. Though being a fatal disease the further complications of this disease can be ceased if it is diagnosed at a proper time and provided with a correct management.

Dentist can probably play a vital role in early diagnosis, referral and management of OSA. A study has reported that high risk OSA patients have increased palatal vault depth, large tongue and Class 3 and 4 Mallampati scores of uvula^10^. These oral features in addition to simple OSA screening questionnaires^5^ can aid the dentist in screening OSA condition in the early stages and referring the patient to sleep medicine department for further confirmatory diagnosis. Further, if proper training is provided, dentists can even fabricate oral appliances for OSA patients^11^. Therefore, it is essential for the dentists to have a thorough knowledge regarding OSA. However, in the management of OSA, the dentists seem to be underutilized.

Manohar et al.^12^ reports that dentist have acceptable knowledge regarding theoretical part of OSA but not much about its management. In India, the prevalence of OSA is around 13.74%^13^. For India, the guidelines for diagnosis and management of OSA came into existence from 2014 after the consistent meetings of Department of Health Research, Ministry of Health and Family Welfare, Government of India under the guidance of Department of Medicine, All India Institute of Medical Sciences, New Delhi, and support of Indian Council of Medical Research^14^. Recently in India, few courses training the dentists in OSA diagnosis and treatment have been evolved^15^. The dental curriculum does not comprise of any learning objectives for OSA screening and management during the undergraduate training^16^.

Hence to evaluate the present scenario of what dentist know, believe and practice regarding OSA, this present study has been undertaken. The aim of this study was to assess the knowledge, attitude and practice regarding the different domains of OSA amongst the dentists in the dental college of India.

## METHODS

This cross-sectional study was conducted from March to June 2017 in Dr. D.Y. Patil Dental College and Hospital, Pimpri, Pune (India). The sample frame consisted of 224 dentists which were a combination of dental faculty and graduate dentists doing their post-graduation studies. 50% of them willing to participate, were randomly chosen (n=112). The ethical approval [No. DPU/R&R(D)/98(38)/1] was obtained from Institutional Ethical Committee prior to starting the study. Written informed consent was obtained from the participants by briefing them the outline of the study along with the assurance of maintaining the anonymity.

### Questionnaire Development

For formulation of questionnaire (the psychometric properties of the questionnaire are a part of other study), a conceptual framework was prepared by identifying the domains to be included through the literature search. Domains considered were definition of OSA, screening and diagnosis, treatment and referral, general findings, risk factors, dental curriculum, interdisciplinary approach, screening diagnosis and treatment and continuing dental education for OSA.

A pool of 29 questions was prepared (14 on knowledge, 8 on attitude and 7 regarding practice). All the questions were closed ended except one (Q1. What is OSA?). The first questionnaire draft was subjected to face validity by 2 experts (result: no addition or deletion of questions needed). Then, 5 experts were asked to perform content validity. Content validity ratio (CVR) was obtained and only those with minimum of 0.99 CVR were retained, while others were either reframed or eliminated. In the knowledge section, four questions were eliminated and seven were reframed; in attitude section, three questions were eliminated and four were reframed and in practice section, two questions were reframed.

The second draft now, consisted of 23 questions with 12 questions on knowledge, 7 questions on attitude and 4 questions on practice. This was again subjected to content validity. The CVR of all the questions was more than the minimum CVR required to keep the question in the final questionnaire. Final questionnaire is presented in Table 1. This questionnaire was pilot tested on 5 participants. With no changes recommended, the data collection was done by distributing the self-administered questionnaire to the participants of the study. It took 5-7 minutes to complete the questionnaire.

**Table 1:** Definition of OSA

**KNOWLEDGE**	

1.	What is Obstructive Sleep Apnea (OSA)?
_________________________________________________________	
**Screening and Diagnosis**	
2.	Gold standard for diagnosis of OSA
a)	STOP questionnaire
**b)**	**Polysomnography**
c)	Case history
d)	Don't know
	
3.	Can final diagnosis of OSA be made by a dentist?
a)	Yes
**b)**	**No**
c)	Don't know
	
**Treatment and Referral**	
4.	Which is not the correct choice of treatment for OSA?
**a)**	**Mild OSA does not require treatment**
b)	Mild OSA treated with Oral appliance
c)	Moderate to severe OSA treated with CPAP and Orthognathic surgeries
d)	Severe OSA treated with oral Orthognathic surgeries
e)	Don't know
	
5.	Disadvantages of CPAP (continuous positive airway pressure) is that, it causes,
a)	Proclination of maxillary incisors
**b)**	**Retroclination of maxillary incisors**
c)	Proclination of mandibular incisors
	
6.	Who can prescribe oral appliances for OSA patients
a)	Dentist
**b)**	**Sleep physician**
c)	Don't know
	
**General findings**	
**7.**	** OSA is more common among?**
**a)**	**Males**
b)	Females
c)	Both
d)	Don't know
	
8.	Is snoring a symptom seen amongst OSA patient?
**a)**	**Yes**
b)	No
c)	Don't know
	
9.	Does the prevalence of OSA increases with age?
**a)**	**Yes**
b)	No
c)	Don't know
**Risk factors**	
10.	Factors which contribute to OSA are
a)	**Obesity**
b)	Hypertension
c)	Obesity and/or hypertension
d)	Don't know
11.	Do you think abnormal maxilla and mandibular development can be a risk factor for OSA?
**a)**	**Yes**
b)	No
c)	Don't know
12.	Enlarged adenoids are risk factors for OSA.
**a)**	**Yes**
b)	No
c)	Don't know
**ATTITUDE**	
**Screening and diagnosis**	
13.	Dentist plays a role in diagnosing and providing treatment for OSA.
**a)**	**Agree**
**b)**	**Strongly agree**
c)	Neutral
d)	Disagree
e)	Strongly disagree
	
14.	When dentist identifies bruxism habit in his patient it is his role to enquire about snoring and OSA.
**a)**	**Agree**
**b)**	**Strongly agree**
c)	Neutral
d)	Disagree
e)	Strongly disagree
	
15.	Is it important for the dentist to enquire about sleep pattern of his patient during history taking?
**a)**	**Agree**
**b)**	**Strongly agree**
c)	Neutral
d)	Disagree
e)	Strongly disagree
	
**Dental curriculum**	
16.	Do you think during under graduation the dental curriculum should include information about OSA and role of dentist?
**a)**	**Agree**
**b)**	**Strongly agree**
c)	Neutral
d)	Disagree
e)	Strongly disagree
	
17.	Should OSA screening of patient be a mandatory part of clinical examination for the dentists.
**a)**	**Agree**
**b)**	**Strongly agree**
c)	Neutral
d)	Disagree
e)	Strongly disagree
**Interdisciplinary approach**	
18.	Dentist and sleep physicians should deal together with OSA patients
**a)**	**Agree**
**b)**	**Strongly agree**
c)	Neutral
d)	Disagree
e)	Strongly disagree
19.	If the dentist encounters abnormal anatomical oral structures then he should further investigate for OSA and refer the patient to sleep physician.
**a)**	**Agree**
**b)**	**Strongly agree**
c)	Neutral
d)	Strongly disagree
e)	Disagree
**PRACTICE**	
**Screening, diagnosis and treatment**	
20.	Have you ever asked your patient about sleep history after observing attrition of teeth in his mouth?
**a)**	**Yes**
b)	No
	
21.	Have you ever screened patient for OSA who has given history of snoring?
**a)**	**Yes**
b)	No
	
I)	If Yes,
**How have you screened the patient for OSA?**	
___________________________________________________________	
	
II)	Have you referred your patient to physician for sleep disordered diagnosis after noticing oral findings related to OSA?
**a)**	**Yes**
b)	No
	
III)	Have you ever fabricated any oral appliance for treating your patient with OSA?
**a)**	**Yes**
b)	No
22.	Have you ever attended any course on management of OSA patients?
**a)**	**Yes**
b)	No
	
23.	Would you be interested in attending course on dental management of sleep related oral diseases?
**a)**	**Yes**
b)	No

### Scoring of Questionnaire

Each correct response was given a score of 1 and incorrect or unanswered were scored 0. The percentage of correct responses was calculated separately for the knowledge, attitude and practice section. To interpret the results domain wise, the total percentage of dentists answering the question correctly were clubbed. Interpretations of the results are as follows:

Good knowledge for a domain: ≥50% dentists giving correct responses; Poor knowledge for a domain: <50% dentists giving correct responses.

Favorable attitude towards a domain: 50% responding to agree and strongly agree; Unfavorable attitude for a domain : <50% dentists responding to neutral, disagree and strongly disagree.

Good practice: ≥ 50% dentists practicing OSA related conditions; Poor practice: <50% dentists not practicing OSA related conditions.

### Analysis

Descriptive analysis were done in the form of percentages for each included question as well as for domain and presented in the form of tables and graphs.

## RESULTS

112 dentists (males=49, females=63) with a mean age of 37.5 years completed the study. The Table 2 depicts the distribution of dentist percentage according to the correct knowledge for each question. More than 50% dentists correctly responded for question regarding, symptoms of OSA (Q.No.8), risk factors for OSA (Q.No.11,12), prevalence of OSA with age (Q.No.9), gender predilection (Q.No.7), disadvantages of CPAP (Q.No.5) and about correctly defining OSA (Q.No.1) Poor knowledge was reported regarding; methods of diagnosing OSA (Q.No.2,3), risk/contributing factors for OSA (Q10) and treatment modalities (Q.No.4,6).

**Table 2:** Frequency distribution of dentists as per their knowledge regarding obstructive sleep apnea (OSA).

Question number	Knowledge		Interpretation
	Correct		
	**(n)**	**(%)**	
1	68	60.71	Good
2	45	40.18	Poor
3	42	37.50	Poor
4	36	32.14	Poor
5	73	65.18	Good
6	12	10.71	Poor
7	71	63.39	Good
8	102	91.07	Good
9	83	74.11	Good
10	38	33.92	Poor
11	87	77.68	Good
12	100	89.29	Good

**Graph 1:**
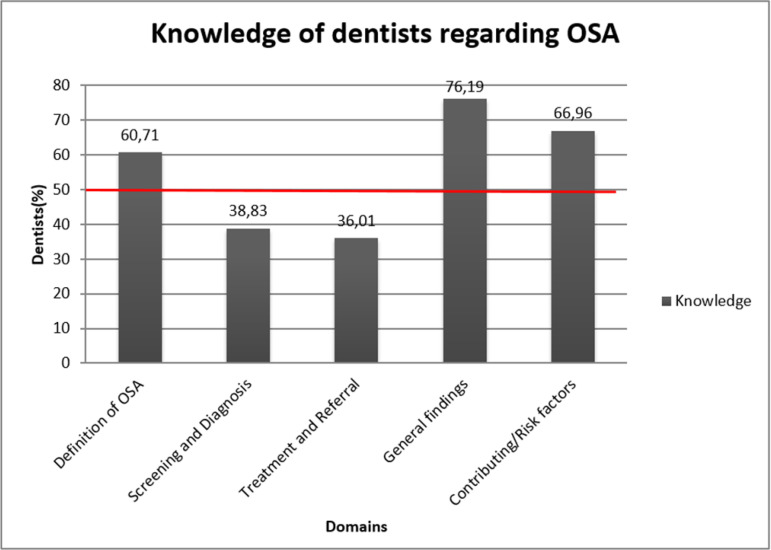
Dentists knowledge based on obstructive sleep apnea (OSA) domains.

Overall, good knowledge amongst the dentists was found for domains regarding, definition, general findings and risk factors of OSA while domains on screening and diagnosis and treatment and referral of OSA cases showed poor knowledge (Graph 1).

**Graph 2:**
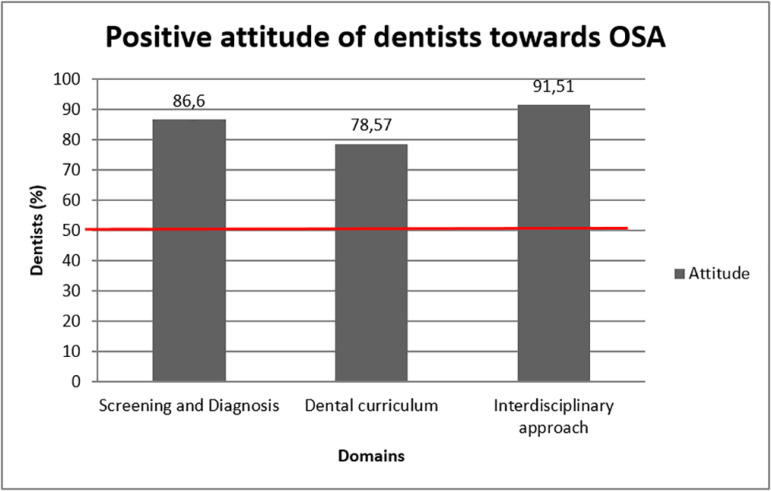
Attitude of dentists based on obstructive sleep apnea (OSA) domains.

100% dentists had a favorable attitude regarding role of dentist in treatment of OSA (Q.No.13), incorporating OSA related knowledge in dental curriculum to undergraduates (Q.No.16), in referral of the patients after identifying relevant oral findings (Q.No.14,19), screening of patients coming to clinics (Q.No.17), enquiring about patient’s sleep pattern (Q.No.15) and interdisciplinary approach (Q.NO.18) (Table 3). Overall, dentists presented favorable attitude for all the domains regarding, screening and diagnosis, dental curriculum and interdisciplinary approach (Graph 2).

**Table 3:** Frequency distribution of the dentists as per their attitude towards obstructive sleep apnea (OSA).

Question number	Attitude		Interpretation
	Favorable		
	(n)	(%)	
13	106	94.64	Favorable
14	95	84.82	Favorable
15	90	80.36	Favorable
16	99	88.39	Favorable
17	77	68.75	Favorable
18	106	94.64	Favorable
19	99	88.39	Favorable

Good practice among dentists was reported for two questions, regarding dentists contribution in asking the patients about sleep history after noticing attrited teeth (Q.No.20) and their interest in attending OSA management courses (Q.No.23) While the other two questions based on screening the patients with snoring history (Q.No.21) and their record on attending OSA management sessions in past (Q.No.22) were responded poorly (Table 4).

**Table 4:** Frequency distribution of the dentists as per their practice regarding obstructive sleep apnea (OSA).

Question number	Practice		Interpretation
	Good		
	(n)	(%)	
20	70	62.50	Good
21	29	25.89	Poor
22	12	10.71	Poor
23	96	85.71	Good

**Graph 3:**
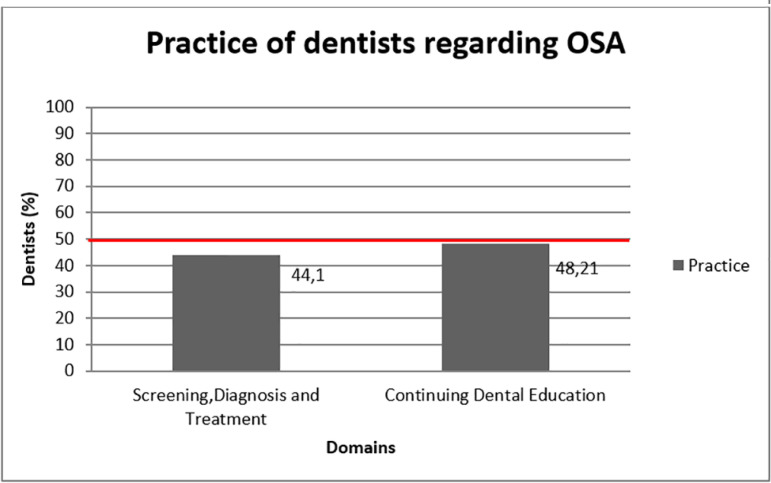
Distribution of dentists according to the good practice regarding obstructive sleep apnea (OSA).

25.89% dentists reported to have screened OSA patients. These dentists had performed screening by observing enlarged mandible, by taking detail history, asking the spouse regarding snoring habits of the patient, measuring neck circumference, through clinical examination, by airway analysis, through sleep test and by discussing the case with a sleep physician (Q No. 21 i). Further, 58.6% referred the screened patients (Q.No.21 ii), while only 44.8% among them fabricated oral appliances for the screened OSA patients (Q.No.21 iii). Largely it was found that more than 50% dentists had poor practice in the domains concerning screening, diagnosis, treatment and continuing dental education (Graph 3).

## DISCUSSION

Of the five domains in the knowledge section the dentists fared poorly for the two domains which were about screening and diagnosis and treatment and referral. The reason for this could be that the dental curriculum does not incorporate learning objective for OSA^16^ and hence the dentist would not have enough knowledge of the same. 60.71% dentists were able to correctly define OSA. Similar kind of result was found among the dentist in the study wherein 75% of the dentist identified the correct definition of OSA from the given options^17^.^( )^

A study reported that around 88% of the dentists could correctly identify the right definition of OSA from the provided options^12^. When it came to OSA and gender predilection in general findings domain, 36.61% dentists were not aware about its relation which was again in accordance with Manohar et al.^12^, while Jokubauskas et al.^18^ reported that around 68.9% dentists correctly reported the relation between gender and OSA.

The results indicate that there is lack of knowledge regarding screening, diagnostic aids, risk factors of OSA as well as a proper treatment of OSA along with the correct time and condition for referral to the sleep physician; regarding their role in final diagnosis and that they themselves cannot prescribe oral appliances to OSA patients directly. Similar kind of result was reported in a study^12^ wherein, 60% of the dentists were not aware about the oral appliances to treat OSA and 21.15% of the dentists could not identify different tests which are carried out for diagnosing OSA.

According to the recommendation of American Academy of Dental Sleep Medicine^3^, the dentist can refer the patient who is suspected of having OSA to a sleep physician who will further refer the patient to undergo sleep test (polysomnography) to arrive at a final diagnosis; the sleep physician is the one who determines whether OSA can be resolved by simple oral appliance or may require a surgical approach; if only oral appliance can solve the issue then sleep physician prescribes dentist to fabricate oral appliance for the OSA patient.

In the present study, the dentists showed a favorable attitude towards domain for screening and diagnosis, dental curriculum and interdisciplinary approach. Within the screening and diagnosis domain, majority of the dentists agreed that dentist and sleep physician should together deal with OSA patients and believed that they too play a major role in diagnosing and providing treatment to OSA patients Similar results were found wherein, 96% of the dentists agreed that it’s a team work of dentist and sleep physician to deal with OSA condition and 75% of the dentists strongly agreed that they can diagnose and provide OSA treatment to the patients^17^.

The results of the present study were also in accordance with Jokubauskas et al.^18^, who reported that 78.8% dentists gave a positive opinion that they and medical practitioner can together deal with OSA. Moreover 41% dentist agreed of their duty for suspecting OSA cases in their clinic and 70.9% gave a positive opinion on participating in OSA treatment^19^. With favorable attitude of dentists for leaning about OSA during graduation it is strongly recommended that the dental curriculum should include topics on OSA.

In the present study, it was observed that the practices of the dentists are poor regarding the domains directed towards screening, diagnosing and treating the OSA patients in their routine dental practice and continuing dental education. Bian^17^ reported that 46% of the dentists refer their patients to sleep physician after suspecting for OSA while Manohar et al.^12^ reported that only 4% dentist diagnosed the OSA patients correctly. 85% dentist never consulted sleep physicians for the suspected OSA cases in their clinics and 89.8% dentists never fabricated any oral appliances for their OSA patients which is similar to the present study^18^.

In the present study, only 10.71% dentists attended extra learning course regarding OSA management and 85.71% dentists expressed their wish to attend courses on OSA management. The results are comparable with Barnes et al.^20^, wherein 90% of the dentists showcased their interest in learning more about OSA while they are contrast with Jokubauskas et al.^18^, who conveyed that 47.3% dentists attended continuing dental education courses for OSA learning.

The present study has been conducted among small sample of the university dentists which can be a limitation because the knowledge level might not be same as compared to the private practice clinicians and thus may not be generalizable. Therefore it is recommended that future studies need to be conducted on a large sample and on the cohort of private practice clinicians.

## CONCLUSION

Overall, it can be postulated that, if a dentist does not have good knowledge about the screening, treatment options and referral system then, he may not be able to correctly diagnose the patient. This may lead to patients going unnoticed. Since OSA is a treatable condition the dentist could potentially play an important role in early identif ication and referral of OSA patients by simply including enquiry of snoring and sleep apnea in the case history proforma and routinely screening for oral findings of OSA patients. Dentist can thus make a significant contribution to the reduction of serious medical symptoms associated with OSA. To further improve the knowledge among the future dentist, it is essential to include OSA topic in undergraduate level itself. Moreover, dentists are required to direct themselves towards continuing dental education programs regarding OSA.
